# Exercise, mitochondrial stress, and trained immunity: metabolic adaptation of innate immunity

**DOI:** 10.3389/fimmu.2026.1902029

**Published:** 2026-07-09

**Authors:** Enli Xie, Yushan He, Yongjun Sun

**Affiliations:** 1 School of Sports Training, Nanjing Sport Institute, Nanjing, China; 2 School of Physical Education and Humanities, Nanjing Sport Institute, Nanjing, China

**Keywords:** DAMPs, epigenetic reprogramming, exercise immunology, metabolic reprogramming, mitochondrial stress, trained immunity

## Abstract

Traditionally recognized as “cellular powerhouses”, mitochondria have gained relevance as pivotal nodes in the integration of metabolism, stress signaling, and innate immunity. In this context, the present work seeks to answer the following question: Does the continuous, exercise-induced mitochondrial stress contribute towards training of innate immune cells by promoting the generation of DAMPs such as mtDNA and succinate? Exercise can be considered as a form of controllable mitochondrial stressor. Mechanistically, the temporary release of mtDAMPs through exercise results in activation of pattern recognition receptors (NLRP3, TLR9, cGAS-STING). Subsequently, there is a metabolic reprogramming event favoring switch from oxidative phosphorylation to aerobic glycolysis along with epigenetic changes (H3K4me3, H3K27ac) priming pro-inflammatory genes for enhanced secondary response. Moderate-intensity exercise develops an immune homeostatic condition with reduced low-grade inflammation and increased reactivity, while sedentary behavior fosters chronic low-grade inflammation, and excessive high-volume exercise can temporarily reduce immune competency. Herein, we present an integrative model where exercise-induced mitochondrial stress as a physiological “training vaccine” to enhance immune surveillance via trained immunity principles. The current model helps differentiate the immune status of elite athletes from sedentary subjects and paves the way for understanding the immunological benefit of exercise prescription in infection prevention, metabolic health, and cancer immunotherapy.

## Introduction

Trained immunity is defined as a reprogramming process in innate immune cells for extended periods of time, stimulated initially by pathogens or endogenous stimuli, leading to augmented responses to secondary stimuli (1). As opposed to adaptive immune memory, which involves gene rearrangements and selection of clones, trained immunity occurs due to changes in metabolism and the epigenome of the cell, making inflammation genes more sensitive to transcriptional activation (2, 3). Trained immunity holds great significance for host defense, both from the point of view of defending against reinfection, as well as chronic inflammatory diseases due to aberrant trained immunity (4).

The identification of trained immunity offers some novel perspectives on how physiological stressors, especially exercise, may impact the activity of the innate immune system. It has become clear that exercise is capable of influencing immune equilibrium in a significant manner: regular, moderate intensity training can lead to a reduction in upper respiratory infection risks by 20–30%, whereas being inactive and exercising too much makes one more vulnerable to infections (5, 6). The relationship between exercise intensity and increased risk of infection has been well studied, but the underlying cellular and molecular mechanisms remain incomplete understood.

Recent developments in mitochondrial biology have indicated that mitochondria act not only as energy suppliers but also as central signaling hubs responsible for coordinating metabolic, stress, and innate immune responses (7, 8). They represent the sites for the formation of inflammasomes, sources of DAMPS like mtDNA and ROS, and mediators of metabolic rewiring during immune cells activation (9). The secretion of mtDAMPs in response to cellular stress activates the signaling cascades mediated by PRRs, including NLRP3 inflammasome activation, TLR9 engagement, and cGAS-STING induction (10, 11).

Given that exercise can generate controlled, temporary oxidative stress within mitochondria, it is conceivable that such a physiological stimulus could be optimal for trained immunity. Acute exercise induces mitochondrial oxidation, ROS production, mtDNA transcription and replication, and even transient mtDAMP release (12). While direct experimental evidence demonstrating that exercise induces classical trained immunity through mitochondrial stress remains limited, the existing data from related fields provide a compelling basis for this hypothesis. Repeated exercises might result in a cumulative reprogramming of the innate immune cells via metabolic and epigenetic processes, generating a state of “immune homeostasis,” where there is low baseline inflammation but increased reactivity to external stimuli (13). On the other hand, individuals who lead a sedentary lifestyle have chronic low-grade inflammation, while high-intensity exercises may impair mitochondrial quality control, leading to maladaptive inflammation.

The purpose of the following literature review is to outline the state-of-the-art research that links exercise, mitochondrial stress, and trained immunity together. First, the concept of trained immunity will be explained along with its mechanisms and metabolic and epigenetic aspects. Secondly, mtDAMPs and their function as immune training triggers will be considered in detail, especially the role of mtDNA and succinate. The next step will be a discussion about how mitochondrial stress associated with physical exercises may contribute to trained immunity activation, with regard to dose dependence of particular types of training. Lastly, these concepts will be put together to draw conclusions on how regular physical activities influence immune system of an individual compared to sedentary one.

## Trained immunity: conceptual and mechanistic foundations

### Definition and historical context

Trained immunity was discovered after noticing that some vaccines like BCG have non-specific effects on unrelated pathogens other than the one they have a target effect on (14). The term trained immunity was introduced in 2011 by Netea et al., who observed an enhanced state gained by innate immune cells following stimulation by bacteria (1). This is seen when stimulated monocytes produce more cytokines compared to unstimulated ones, with this enhanced state persisting for several weeks after the clearance of the initial stimulus.

Trained immunity is characterized by: i) activation through the action of an initial stimulus, which can be microbial, endogenous, or nutritional; ii) persistent functional enhancement; iii) cross-protective activity against unrelated stimuli; and iv) reprogramming of metabolism and epigenetics without genetic alteration (2, 3). These characteristics are what set trained immunity apart from other types of innate immune memory, such as tolerance, where the response becomes weaker, or priming, where short-term activation occurs (15).

### Metabolic reprogramming as the foundation of trained immunity

One of the key hallmarks of trained immunity is the dramatic change in cellular metabolism associated with the induction of immunity. At rest, macrophages and monocytes utilize OXPHOS to generate ATP; however, following activation, there is a metabolic reprogramming towards aerobic glycolysis, similar to that which occurs in cancer cells, known as the Warburg effect (16).

Cheng et al.’s study showed that induction of trained immunity in monocytes by β-glucan is characterized by enhanced glycolysis and impaired OXPHOS (17). Blockade of glycolysis inhibited trained immunity, whereas disruption of OXPHOS failed to exert an impact on the phenomenon. Hence, metabolic shift toward aerobic glycolysis was shown to be critical for the induction of trained immunity.

Later, Arts et al. presented a detailed overview of the metabolic pathways underlying trained immunity utilizing transcriptomic and metabolomic analyses (18). In addition to glycolysis and OXPHOS, they demonstrated that glutaminolysis and the cholesterol synthesis pathway play vital roles in the process. Glutamine metabolism leads to an increase in the intracellular pool of fumarate, a product of glutamine metabolism and a key signaling molecule for the induction of trained immunity. The use of glutaminase and HMGCR inhibitors (statins) blocked trained immunity induction.

Metabolic rewiring towards glycolysis plays dual roles as follows (**Table 1**); first, it ensures rapid production of ATP that powers the increased function of effectors, and second, it produces substrates needed for epigenetic changes (19). Acetylation of histones, an important epigenetic mark involved in trained immunity, depends on the availability of acetyl-CoA, which is a product of glycolytic pathway. Metabolism and epigenetics therefore go hand in hand in establishing trained immunity.

**Table 1:** Metabolic pathways in trained immunity.

Metabolic pathway	Role in trained immunity	Key enzymes/mediators	Inhibition effect
Glycolysis	Provides rapid ATP and biosynthetic precursors for epigenetic modifications; essential for trained immunity induction	HIF-1α, mTOR, hexokinase, PFKFB3	Blocks trained immunity establishment
Glutaminolysis	Supplies carbon skeletons for TCA cycle intermediates; promotes fumarate accumulation	GLS1, GOT1	Attenuates trained immunity
Cholesterol synthesis	Modulates inflammatory gene expression via mevalonate pathway	HMGCR, mevalonate	Abrogates trained immunity
OXPHOS	Maintained at low levels; reduced flux through SDH contributes to succinate accumulation	SDH, ETC complexes	Not sufficient for trained immunity

### Epigenetic mechanisms of trained immunity

Though metabolic reprogramming forms the source of energy for the trained state, epigenetic regulation serves as a memory component that allows sustained maintenance of the trained state. As opposed to adaptive immune memory, which depends on genomic rearrangements, trained immunity makes use of changes in the chromatin structure, which are permanent yet reversible (20).

A defining feature of epigenetic regulation in trained immunity is the presence of activating histone modifications in the promoter regions of pro-inflammatory genes such as trimethylation of lysine 4 of histone H3 (H3K4me3) and acetylation of lysine 27 of histone H3 (H3K27ac) (21). Such histone modifications serve to prime the genes for quick activation. Saeed et al. showed that β-glucan exposure results in H3K4me3 in pro-inflammatory genes in monocytes, and such modifications are maintained even after removal of the stimulus (22).

HDACs have an important role in the development of trained immunity through controlling the state of acetylation of both histones and other proteins. SCFAs including butyrate are inhibitors of HDACs and induce trained immunity-like effects (23). Training has been demonstrated to affect the activity of HDACs in immune cells. This could explain the relationship between exercise and trained immunity through epigenetics (24).

The relatively stable DNA methylation is another type of epigenetic modification that may be related to the persistence of trained immunity. According to recent studies, the DNA methylation pattern of trained monocytes at inflammatory genes is altered and persists for extended periods. This is the source of memory that is passed from mother cells to daughter cells (25). Changes in DNA methylation due to exercise have also been reported in immune cells, although their relation to trained immunity requires further investigation (26). **Table 2** provides information about epigenetic modifications involved in trained immunity.

**Table 2:** Epigenetic modifications in trained immunity.

Epigenetic mark	Role in trained immunity	Associated genes	Persistence duration
H3K4me3	Poises promoters for rapid transcription upon restimulation	TNF, IL6, IL1B	Days to weeks
H3K27ac	Enhances transcriptional activation	IL12, NOS2	Days to weeks
H3K27me3	Repressive mark; reduced in trained monocytes	SOCS3, IL10	Days
DNA hypomethylation	Stable memory mechanism enabling persistence through division	Promoter regions of inflammatory genes	Weeks to months
Histone lactylation	Links glycolysis to histone modifications	Multiple genes	Under investigation

## Mitochondrial DAMPs: initiators of innate immune training

### Mitochondria as sources of DAMPs

Mitochondria are enclosed in double membranes and are primarily known to perform functions related to energy production, calcium buffering, and apoptosis (**Table 3**). Recently, however, an increasing number of studies have confirmed that mitochondria function as central signaling hubs linking cellular metabolism, stress responses, and innate immunity (7, 8). Upon damage or stress exposure, mitochondria secrete several types of signals, which are perceived by the host as non-self and thus named mtDAMPs (mitochondrial damage-associated molecular patterns) (27, 28).

**Table 3:** Dose-dependent effects of exercise on trained immunity outcomes.

Exercise modality	Mitochondrial stress	mtDAMP release	Training effect	Immune outcome
Sedentary (none)	Low/absent	Low basal levels	None	Chronic low-grade inflammation
Moderate-intensity continuous training	Controlled, transient	Moderate, transient	Mitochondrial biogenesis enhanced; OXPHOS efficiency increased; trained immunity established	Immune homeostasis; reduced infection risk
High-intensity interval training	High, episodic	High, transient	Mixed effects; may transiently overwhelm MQC	Enhanced acute responses; potential overtraining with excessive volume
Prolonged exhaustive exercise	High, sustained	High, sustained	MQC overwhelmed; mtDAMP accumulation	Transient immune suppression; increased infection risk

Important examples of mtDAMPs include mtDNA, which is a CpG-rich, circular molecule that resembles bacterial DNA and is involved in the activation of TLR9 and cGAS-STING pathways (29); mtROS, which are produced during OXPHOS and have the capacity to activate the NLRP3 inflammasome and produce oxidative stress (30); succinate, a molecule that builds up during inflammation, stabilizes HIF-1α, and stimulates IL-1β synthesis (31); ATP, which upon secretion from damaged mitochondria, activates purinergic receptors and triggers inflammatory pathways (32); cardiolipin, a phospholipid specific to mitochondria and an activator of the NLRP3 inflammasome (33); and transcription factor A, mitochondrial (TFAM) (34).

mtDAMPs are secreted by multiple pathways, which include pore formation through BAX/BAK, mPTP opening, and GSDMD pore formation after inflammasome activation (35). After their secretion to the cytosolic or extracellular milieu, mtDAMPs bind different PRRs, initiating signal transduction that ultimately controls innate immune reactions.

### mtDNA and the cGAS-STING pathway

A key role of mitochondrial DNA release was shown by Shimada et al., where oxidized mtDNA released during apoptosis binds the NLRP3 inflammasome complex leading to caspase-1 activation and IL-1β/IL-18 secretion (10). Thus, mitochondria contribute to sterile inflammation in many ways, from the generation of mitochondrial ROS to mtDNA release and sensing by different pattern recognition receptors.

As the cGAS-STING pathway is an important receptor of cytosolic DNA, a key player in mtROS signaling was found to be cyclic GMP-AMP synthase (cGAS), which binds double-stranded cytosolic DNA and generates cGAMP that further binds STING to activate its signaling resulting in type I interferon responses and inflammatory cytokines secretion (36). Moreover, mtDNA can trigger the cGAS-STING axis in response to various stressful events, including exercise-induced stress (37).

Importance of mtDNA release was confirmed in different pathological processes. The authors of (11) have found that formylated mtDNA released after trauma activates neutrophils via TLR9 and leads to systemic inflammatory response syndrome. In addition, recent reports suggest involvement of mtDNA release in the aging-associated inflammatory response when accumulation of cytoplasmic mtDNA activates cGAS-STING signaling leading to cellular senescence (38).

### Succinate as a mitochondrial signaling molecule

Succinate is an intermediate of the citric acid cycle pathway, whose concentration rises when mitochondria undergo stress due to inhibited succinate dehydrogenase (SDH) activity or when the citric acid cycle is disrupted at SDH through metabolic changes associated with inflammation (31). An increase in glycolytic flux results in accumulation of citric acid cycle intermediates like succinate in inflamed macrophages, promoting IL-1β production (39).

As described by Mills et al., succinate accumulation within activated macrophages is essential for the induction of inflammation-driven genes (31). In this respect, succinate behaves not only as an intermediary in the metabolism pathway, but also as a signaling molecule whose activity depends upon the stabilization of HIF-1α and activation of its downstream signaling via the succinate receptor (SUCNR1) (40).

This importance of succinate signaling in the context of exercise is highlighted by evidence indicating that succinate is secreted from contracting muscle fibers in response to exercise and activates the SUCNR1 receptor on immune cells (41). Recent studies conducted by Reddy et al. revealed that M2-polarized macrophages were sensitive to the actions of extracellular succinate via the SUCNR1 receptor (42).

**Figure 1:**
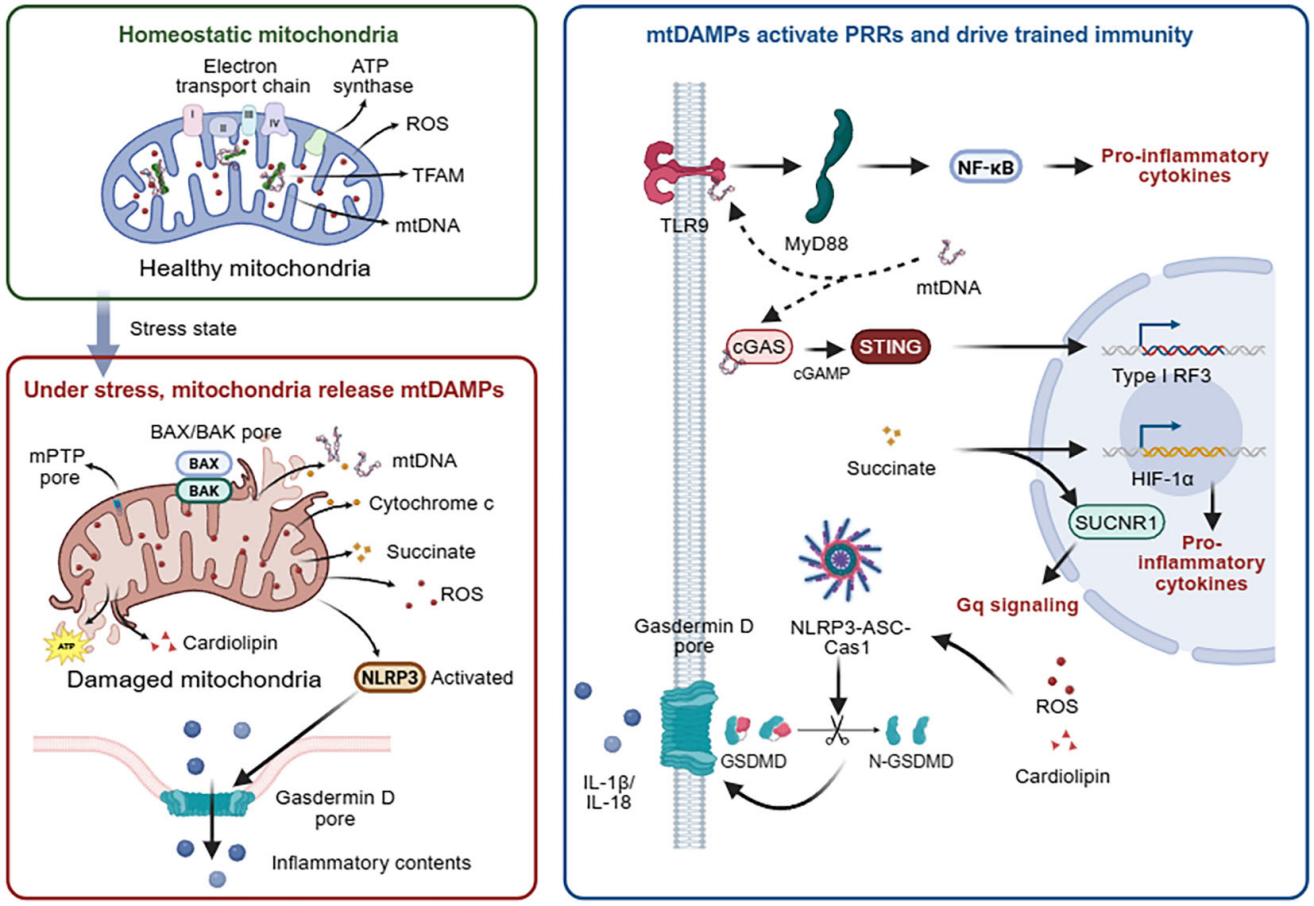
Mitochondrial DAMPs in trained immunity: Mechanisms of secretion and signaling pathways. **(A)** Under normal physiological conditions, mitochondria ensure ATP generation through OXPHOS while maintaining tight regulation of mitochondrial DNA (mtDNA) packing by TFAM, along with well-regulated generation of ROS. **(B)** In stress conditions, such as exercise, mitochondria export mtDAMPs using different routes: BAX/BAK pores, mitochondrial permeability transition pore (mPTP), and GSDMD pores upon inflammasome activation. Some of the mtDAMPs are mtDNA fragments, succinate, ROS, ATP, and cardiolipin. **(C)** The activation of pattern recognition receptors is accomplished by the binding of mtDAMPs to their respective receptors: mtDNA binds to TLR9 and cGAS-STING, while succinate stabilizes HIF-1α and activates SUCNR1/GPR91, ROS and cardiolipin activate NLRP3 inflammasome.

A diagrammatic representation of mitochondrial DAMP secretion mechanisms and their activation of innate immune signaling cascades is presented in **Figure 1** below.

## Exercise as a controllable mitochondrial stressor

### Exercise-induced mitochondrial adaptations

Exercise is among the most effective physiological stimulus of mitochondrial adaptations (43). Skeletal muscle comprises about 40% of the body weight, and this tissue features a dynamic network of mitochondria, which are sensitive to both short- and long-term exercise and undergo morphological and functional adaptations accordingly.

In response to acute exercise, mitochondrial respiration and ROS generation increase depending on exercise intensity (44). This short-term change is followed by the increase in mtDNA transcription and replication processes, resulting in an increase in mtDNA copies (45). Furthermore, the energy demands associated with exercise stimulate mitochondrial dynamics with a predominance of fission, which leads to defective mitochondria elimination through mitophagy (46).

Chronic exercise training stimulates mitochondrial biogenesis via stimulation of the PGC-1α pathway, which results in increased mitochondrial content and greater oxidative capacity (43). The mitochondria of trained people are characterized by an increased density and more effective functioning of the respiratory electron transport chain and, hence, decreased generation of ROS both at rest and during submaximal exercise. Such adaptations can help mitigate the effects of chronic inflammation that accompany a sedentary lifestyle (47).

It is crucial to note that the intensity and time of exercise influence the type of mitochondrial stress caused. While moderate-intensity continuous training(MICT)leads to improved mitochondria quality regulation and better energy efficiency without increasing oxidative stress, high-intensity interval training(HIIT)and exhaustive exercise overload the quality control of mitochondria, thus inducing the leakage of mtDAMPs into the cytosol and inflammatory reactions (48, 49).

### Exercise-induced release of mtDAMPs

Multiple studies have shown that exercise can lead to the release of mtDAMPs into the bloodstream. Exercise intensity and duration were correlated with elevated amounts of circulating mtDNA (50, 51). Exercise-induced mtDNA release might be related to skeletal muscles as well as immune cells present in the bloodstream.

As Rodrigues et al. found out quite recently, the acute response to exercise results in proportional elevation of circulating cell-free DNA (cfDNA) from neutrophils, dendritic cells, and macrophages in a dependence on exercise intensity (52). Of note is the fact that training attenuated cfDNA release during HIIT in addition to reduction of cfDNA release from dendritic cells and macrophages (not neutrophils).

The precise mechanism through which the mtDNA is released by exercise is unknown but includes mPTP opening, formation of BAX/BAK-mediated pores, and formation of GSDMD pores subsequent to the activation of NLRP3 inflammasome (35). The temporary nature of elevated mtDAMPs after exercise is a distinguishing factor because it contrasts with the persistent elevated levels associated with pathologies such as chronic inflammation.

The mitochondrial metabolite succinate is released by contracting muscles during exercise and acts as an energy source (53, 54). Exercise-induced succinate release could have two purposes; stimulating SUCNR1 receptors in immune cells to regulate inflammatory responses, and acting as a substrate for stabilizing HIF-1α in trained macrophages.

### Exercise, mitochondrial quality control, and NLRP3 regulation

The mitochondrial quality control(MQC)processes like mitochondrial biogenesis, fusion/fission, and mitophagy play a vital role in preserving mitochondrial integrity and prevent the leakage of mtDAMPS (55). Exercise is potent in the activation of MQC and enhances the clearance of dysfunctional mitochondria and renews the pool of mitochondria (56).

Tran et al. explored the influence of aging, endurance exercise, and denervation on the innate immune response in skeletal muscle (57). Their findings revealed that denervation causes loss in muscle mass, mitochondrial content, and impaired respiration along with increased expression levels of NF-κB p65 and inflammatory mediators such as NLRP3, caspase-1, GSDMD-N, STING, and IL-1β. Notably, endurance exercise had a uniform effect of decreasing the age-related increases in inflammatory proteins and showed a tendency towards the lowering of cytosolic mtDNA observed in aged muscle.

Conclusions from a meta-analysis described in Frontiers in Immunology stated that resistance training, aerobic training, and combination training for 8 weeks or longer are associated with a decrease in key NLRP3 inflammasome pathway proteins, which is evidence that exercise is safe and effective in controlling NLRP3-mediated inflammation (58).

Adaptations of mitochondrial quality control processes and their interaction with innate immune signaling pathways in skeletal muscle were discussed comprehensively by Brestoff et al. in a narrative review, concluding that modulation of skeletal muscle activity may be a useful treatment for inflammation-related muscle disorders due to exercise training.

### Dose-dependent effects of exercise on innate immune training

Exercise intensity-immune system health interaction is J-shaped, which implies that sedentary people have moderate risks of infections; those involved in moderate exercises have their infection risk reduced by 20-30% while high intensity/high volume training increases their infection risks (5, 6). Dose response effects of exercise intensity on immune system health are based on stress and training effects on mitochondria.

Consistent moderate exercise seems to offer the ideal stressor for inducing trained immunity: intense enough to create epigenetic reprogramming effects, yet not too vigorous to exceed the capacity of MQC, leading to inflammation. On the other hand, the state of physical inactivity is linked with chronic low-level inflammation (raised levels of CRP, IL-6, TNF-α), indicating insufficient MQC and inappropriate mtDAMP signaling (60).

## Exercise-induced trained immunity: mechanistic integration

### From mitochondrial stress to innate immune training

The integration of the above evidence suggests a comprehensive model through which repeated moderate intensity exercises train innate immunity via mitochondrial stress signaling. The proposed sequence is as follows:

Step 1: Exercise-induced mitochondrial stress. Each exercise session increases mitochondrial respiration and mtROS generation. This transient stress activates mitochondrial quality control systems including mitophagy.

Step 2: mtDAMP release. Exercise triggers temporary release of mtDNA and succinate via mPTP opening and BAX/BAK pore formation, which are recognized by PRRs on innate immune cells.

Step 3: PRR activation and signaling. mtDNA activates TLR9 and cGAS-STING; succinate stabilizes HIF-1α and activates SUCNR1; ROS and cardiolipin activate NLRP3 inflammasome. These pathways converge on NF-κB activation.

Step 4: Metabolic reprogramming. Activation signals promote a shift from OXPHOS to aerobic glycolysis via HIF-1α, supplying acetyl-CoA and biosynthetic precursors for epigenetic modifications.

Step 5: Epigenetic establishment of trained immunity. Exercise training promotes deposition of H3K4me3 and H3K27ac at pro-inflammatory gene promoters, enhancing responsiveness to secondary stimuli.

This model provides a framework for understanding how repeated moderate exercise sessions may progressively establish a state of trained immunity through cumulative mitochondrial stress signaling.

### Alternative interpretations and evidence gaps

While the proposed model provides a coherent framework, several important caveats warrant consideration. First, it remains unclear whether exercise-induced immune changes represent classical “trained immunity” as defined by Netea et al.—i.e., enhanced secondary responses following a primary stimulus—or whether they reflect other forms of immune modulation such as “priming” (short-term activation) or “adaptation” (non-specific enhancement without epigenetic reprogramming) (2, 15). Direct head-to-head comparisons between exercise-trained immune cells and β-glucan- or BCG-trained cells are lacking.

Second, exercise effects on immunity may be mediated through pathways independent of mitochondrial stress, including neuroendocrine signaling (catecholamines, glucocorticoids), hemodynamic changes, and gut microbiome alterations (47). Disentangling the relative contributions of these pathways remains challenging with current experimental approaches.

Third, the J-shaped relationship between exercise intensity and infection risk may have alternative explanations beyond mitochondrial stress, such as changes in mucosal immunity, salivary IgA levels, or neutrophil function (6, 59).

These alternative interpretations underscore the need for direct experimental validation of the proposed model, as outlined below.

### Hematopoietic stem cell training: central trained immunity

The fact that trained immunity persists from weeks to months presents another question; how is it possible to maintain the training effect when monocytes and macrophages have very short half-lives? Recent research shows that training of immunity takes place due to hematopoietic stem cells(HSC)programming referred to as “central trained immunity” (61).

β-Glucan training leads to stable programming of HSCs with an enhanced capacity of responding, as progeny generated from trained HSCs exhibit higher responsiveness (62). Functional programming is brought about by epigenetic changes that occur within the HSC pool, which are later transferred to the differentiated offspring.

In an article, Kaufmann and co-workers revealed that BCG vaccine training of HSCs produces protective innate immunity to Mycobacterium, the effect of which lasts for months (63). Hematopoietic stem and progenitor cells (HSPC) provide an excellent pool for trained immunity as they undergo epigenetic changes that get transmitted to their progeny (64).

Recently, Tran et al. gave a detailed review on how HSPCs function as the pool for trained immunity (64). According to them, due to the relatively short half-life of mature innate immune cells, it is only possible to have trained immunity maintained in the body by programming at the level of progenitors. It could be postulated that exercise might train HSCs by means of mtDAMP signaling through mitochondrial stress.

### Immune homeostasis: distinguishing the athlete from the sedentary individual

Trained immunity provides a mechanistic framework for distinguishing the immune characteristics of physically active individuals from those who are sedentary. The key differences can be summarized as follows:

Sedentary individuals exhibit low-grade chronic inflammation (elevated CRP, IL-6, TNF-α) with blunted acute responses (65). This likely reflects an imbalance in MQC with continuous low-level mtDAMP production and PRR activation, without the metabolic and epigenetic reprogramming that characterizes trained immunity.

Physically active individuals (moderate training) develop immune homeostasis—low baseline inflammation with enhanced responsiveness to immune challenges (66). This state arises from repetitive mitochondrial signaling that may drive metabolic and epigenetic reprogramming in a non-damaging manner.

Elite athletes during high-volume training may experience immune dysfunction with increased infection rates and elevated inflammatory markers (67, 68), suggesting that excessive training exceeds optimal mitochondrial stress thresholds.

## Implications for clinical practice and future research

### Exercise prescription for immune optimization

This realization of exercise as a mechanism for developing trained immunity can have concrete applications in exercise programming for improved immune health. Based on the current evidence, moderate intensity continuous exercise (30 to 60 minutes, 3 to 5 days a week, at 50 to 70% VO_2_max) is probably the most effective way to elicit trained immunity without compromising immune function through overtraining.

This recommendation agrees with the guidelines set out by public health institutions with regards to exercise for cardiovascular and metabolic disease prevention, thus making exercise a viable approach for immune improvement. Patients with inflammation disorders may require reduced intensities and/or duration, with gradual progression as immune function improves.

### Exercise and vaccination: adjuvant effects

Vaccination and training immunity elicited through exercise may help improve vaccine response efficiency. There is animal evidence that exercise during vaccination improves antibody production as well as T cell response (69, 70). It is still not known whether exercise helps to improve vaccine-trained immunity (which means improved innate responses that help to boost adaptive responses).

The following are some of the ways through which exercise may help improve vaccine response: (i) encouraging lymphocytes to migrate to the lymph nodes; (ii) inducing inflammation that helps to enhance antigen presentation; (iii) improving metabolic health of immune cells; and (iv) training the immune system to react to vaccine adjuvant.

### Exercise in cancer immunotherapy

The notion that trained immunity can boost anti-tumor activities has prompted investigations into exercise as an adjuvant for immunotherapy against cancer (71, 72). Moderate intensity aerobic exercise training boosts effector function of CD8^+^ tumor infiltrating lymphocytes via prevention of mitochondrial depletion and improvement of mitochondrial content and function in T cells (72).

Trained immunity from exercise might promote anti-tumor activities via: (i) improvement in surveillance of innate immunity; (ii) reprogramming of metabolism of tumor-associated macrophages from pro-tumor to anti-tumor; and (iii) enhancement of trafficking of T cells in the tumor environment. Clinical trials assessing exercise in immunotherapy are currently underway.

### Future research directions

Despite rapid progress, several key questions remain unanswered:

Mechanistic causality and direct evidence: Does exercise directly induce classical trained immunity, or are observed immune changes merely correlational? To date, no study has directly demonstrated that exercise-trained immune cells exhibit the hallmark features of trained immunity: enhanced cytokine production upon secondary stimulation, sustained epigenetic modifications (H3K4me3, H3K27ac), and the characteristic metabolic shift toward aerobic glycolysis. Controlled interventional studies with longitudinal assessment of trained immunity biomarkers are urgently needed. Studies comparing exercise-induced immune changes to classical trained immunity inducers (e.g., β-glucan, BCG) would be particularly informative.

Dose-response optimization: What would be the ideal exercise dosage that should be employed for developing trained immunity? How do the three components of intensity, frequency, and duration play together to achieve results?

Tissue specificity: Is exercise able to induce innate immune training differently in various tissues? Tissue-specific responses induced by exercise (for instance, in lung, gut, and adipose tissues) need further study.

Individual variability: What makes people respond differently to their trained immunity after exercising? Genetics, metabolism, and microbiome are some possibilities that may help personalize exercise regimens.

Long-term persistence: For how long does trained immunity persist post-exercise? Is de-training sufficient to reverse this phenomenon, or is there some residual “immune memory” after the process?

Aging and disease: Does exercise increase trained immunity among older individuals and immunosuppressed patients? Can exercise-induced trained immunity be used as an alternative therapeutic target against infection susceptibility without pharmaceuticals?

## Conclusion

The discovery of trained immunity has fundamentally reshaped our understanding of innate immunity, revealing that monocytes and macrophages can develop enhanced responsiveness through metabolic and epigenetic rewiring. Mitochondria have emerged as central hubs integrating metabolism, stress signaling, and innate immune activation. mtDAMPs such as oxidized mtDNA and succinate, generated during exercise-induced mitochondrial stress, activate PRRs and trigger pathways that drive metabolic and epigenetic reprogramming. We propose that repetitive moderate-intensity exercise may establish a trained immunity state.

This framework distinguishes the immune status of regularly exercising individuals (trained immunity-associated immune homeostasis) from sedentary individuals (chronic low-grade inflammation), providing a mechanistic basis for the J-shaped relationship between exercise intensity and infection risk. Recognizing exercise as a physiological mitochondrial stressor that may train innate immunity opens new avenues for exercise interventions in infection prevention, metabolic health, vaccination enhancement, and cancer immunotherapy. Elucidating causative mechanisms, establishing optimal exercise dosing for trained immunity induction, and translating these concepts to clinical practice remain key priorities.

## Glossary

AIM2: Absent in melanoma 2
AMPK: AMP-activated protein kinase
ATP: Adenosine triphosphate
BAX: BCL-2-associated X protein
BCG: Bacillus Calmette-Guérin
BER: Base excision repair
cGAS: Cyclic GMP-AMP synthase
cGAMP: Cyclic GMP-AMP
CMPK2: Cytidine monophosphate kinase 2
CLR: C-type lectin receptor
CRP: C-reactive protein
DAMPs: Damage-associated molecular patterns
DNA: Deoxyribonucleic acid
DRP1: Dynamin-related protein 1
ENDOG: Endonuclease G
ETC: Electron transport chain
GSDMD: Gasdermin D
H3K4me3: Trimethylation of lysine 4 on histone H3
H3K27ac: Acetylation of lysine 27 on histone H3
H3K27me3: Trimethylation of lysine 27 on histone H3
HDAC: Histone deacetylase
HIF-1α: Hypoxia-inducible factor 1-alpha
HIIT: High-intensity interval training
HMGCR: 3-Hydroxy-3-methylglutaryl-CoA reductase
HSCs: Hematopoietic stem cells
HSPCs: Hematopoietic stem and progenitor cells
IFN: Interferon
IL-1β: Interleukin-1 beta
IL-6: Interleukin-6
IL-10: Interleukin-10
IRF3: Interferon regulatory factor 3
MICT: Moderate-intensity continuous training
MQC: Mitochondrial quality control
mPTP: Mitochondrial permeability transition pore
mtDNA: Mitochondrial DNA
mtDAMPs: Mitochondrial damage-associated molecular patterns
mtROS: Mitochondrial reactive oxygen species
mTOR: Mammalian target of rapamycin
NF-κB: Nuclear factor kappa-light-chain-enhancer of activated B cells
NLRP3: NLR family pyrin domain containing 3
OGG1: 8-Oxoguanine DNA glycosylase
OPA1: Optic atrophy 1
OXPHOS: Oxidative phosphorylation
PAMPs: Pathogen-associated molecular patterns
PGC-1α: Peroxisome proliferator-activated receptor gamma coactivator 1-alpha
PGAM5: Phosphoglycerate mutase family member 5
PINK1: PTEN-induced putative kinase 1
PNKP: Polynucleotide kinase/phosphatase
PRRs: Pattern recognition receptors
RAB5: Ras-related protein Rab-5
RAB7: Ras-related protein Rab-7
ROS: Reactive oxygen species
SAM50: Sorting and assembly machinery component 50
SCFAs: Short-chain fatty acids
SDH: Succinate dehydrogenase
SIRT1: Sirtuin 1
STING: Stimulator of interferon genes
SUCNR1: Succinate receptor 1 (also known as GPR91)
TBK1: TANK-binding kinase 1
TCA cycle: Tricarboxylic acid cycle (also known as Krebs cycle)
TFAM: Mitochondrial transcription factor A
TGF-β: Transforming growth factor-beta
TLR9: Toll-like receptor 9
TNF-α: Tumor necrosis factor-alpha
TREX1: Three prime repair exonuclease 1
VDAC: Voltage-dependent anion channel
ZBP1: Z-DNA binding protein 1
